# On Performance Analysis of Protective Jamming Schemes in Wireless Sensor Networks

**DOI:** 10.3390/s16121987

**Published:** 2016-11-24

**Authors:** Xuran Li, Hong-Ning Dai, Hao Wang, Hong Xiao

**Affiliations:** 1 Faculty of Information Technology, Macau University of Science and Technology, Macau, China; 1509853dii30001@student.must.edu.mo; 2 Big Data Lab, Norwegian University of Science and Technology in Aalesund, 6009 Aalesund, Norway; hawa@ntnu.no; 3 Faculty of Computer, Guangdong University of Technology, Guangzhou 510006, China; wh_red@gdut.edu.cn

**Keywords:** security, wireless sensor networks, friendly jamming, analysis

## Abstract

Wireless sensor networks (WSNs) play an important role in Cyber Physical Social Sensing (CPSS) systems. An eavesdropping attack is one of the most serious threats to WSNs since it is a prerequisite for other malicious attacks. In this paper, we propose a novel anti-eavesdropping mechanism by introducing friendly jammers to wireless sensor networks (WSNs). In particular, we establish a theoretical framework to evaluate the eavesdropping risk of WSNs with friendly jammers and that of WSNs without jammers. Our theoretical model takes into account various channel conditions such as the path loss and Rayleigh fading, the placement schemes of jammers and the power controlling schemes of jammers. Extensive results show that using jammers in WSNs can effectively reduce the eavesdropping risk. Besides, our results also show that the appropriate placement of jammers and the proper assignment of emitting power of jammers can not only mitigate the eavesdropping risk but also may have no significant impairment to the legitimate communications.

## 1. Introduction

Cyber Physical Social Sensing (CPSS) has emerged as a promising paradigm to enable the interactions between humans and the physical environment [1,2,3,4]. As a key component of CPSS systems, wireless sensor networks (WSNs) play an important role in sensing, collecting and transmitting confidential information [5,6]. However, WSNs are also susceptible to various malicious attacks due to the vulnerability of sensor nodes [7]. *Eavesdropping* attack, as one of typical malicious attacks in WSNs has attracted considerable attention recently. It is difficult to detect eavesdropping behaviours since *malicious* nodes (also called eavesdroppers) passively wiretap the confidential information without disclosure of their existence.

Encryption has been typically used to protect the confidential communications in wireless networks. For example, Cellular Message Encryption Algorithm has been used in cellular networks [8] and KASUMI has been used in 3G networks [9] while wireless local area networks (WLANs) have adopted Wired Equivalent Privacy (WEP) [10], Wi-Fi Protected Access (WPA) and Wi-Fi Protected Access II (WPA2) [11]. However, the traditional cryptographic methods may not be feasible to WSNs due to the following constraints: 1) the limited battery power of sensor nodes; (2) the inferior computational capability of sensor nodes and (3) the difficulty of managing distributed sensor nodes in a centralized way, which is however necessary for many encryption algorithms [12].

In this paper, we propose a novel anti-eavesdropping mechanism to protect confidential communications in WSNs. In particular, we deploy a small number of *friendly jammers* in WSNs, which can generate sufficient interference to prevent eavesdroppers from snooping legitimate communications. We name such schemes as Friendly-Jamming (Fri-Jam) schemes. Recently, [13,14,15,16,17] also proposed a similar approach named *Protective Jamming* (Pro-Jam) to prohibit the eavesdropping attacks in RFID-like networks. However, Pro-Jam is mainly designed for the environment with a fence at the boundary of the network, where jammers are placed outside the fence. This assumption is impractical to WSNs since eavesdroppers can appear at any location in WSNs. Besides, most of the study on Pro-Jam scheme have been focused on the power assignment in a specific scenario. To the best of our knowledge, *there is a lack of performance analysis on friendly-jamming schemes*.

In this paper, we establish a general analytical model to evaluate the performance of Fri-Jam schemes. The primary contributions of this paper can be summarized as follows.

- In particular, we propose a general theoretical model to quantify the eavesdropping risk (measured by the eavesdropping probability) and evaluate the impact of Fri-Jam schemes on the legitimate communications (measured by the transmission probability).
- We consider three types of Fri-Jam schemes: random placement of jammers (named FJ-Ran scheme), regular placement of jammers (named FJ-Reg scheme) and FJ-Reg scheme with power control (named FJ-PC scheme).
- We compare the eavesdropping probability of WSNs without jammers with that with friendly jammers (FJ-Ran, FJ-Reg and FJ-PC schemes). We find that all of three Fri-Jam schemes can effectively reduce the eavesdropping probability in contrast to no-jamming scenarios.
- Our results also show that the appropriate placement of friendly jammers in WSNs can significantly reduce the eavesdropping probability whilst there is no significant impairment on legitimate communications. Besides, to adjust emitting power of jammers properly can mitigate the eavesdropping risk while has no significant impairment to the legitimate transmission.

The rest of this paper is organized as follows. We summarize the related works in Section 2. Section 3 introduces the models used in this paper. We then analyze the eavesdropping probability of different Fri-Jam schemes in Section 4. We next show the results in Section 5. Finally, Section 6 concludes this paper.

## 2. Related Work

It is difficult to detect eavesdropping attacks in WSNs since eavesdroppers passively snoop the confidential communications with concealment of their presence. Encryption is one of the most commonly used techniques to protect confidential communications, which is shown to work effectively in WLANs (e.g., WEP [10], WPA and WPA2 [11]), in cellular networks (e.g., CMEA [8] and KASUMI [9]) and in wireless personal area networks (WPANs) [18]. However, applying such cryptography-based techniques help hiding the meaning of the information being transmitted, but not the existence of the information itself. In addition, the techniques are designed to make it computationally difficult for the adversary to understand the true meaning of the information while the adversary is still able to access all the information [19]. Furthermore, it is quite challenging to apply the conventional ciphers (encryption algorithms) to WSNs due to the following inherent constraints of WSNs [12]: (a) the inferior computational capability of wireless nodes; (b) the limited battery power of wireless nodes; (c) the difficulty of managing the distributed sensor nodes in the centralized manner. In addition, the cryptographic authentication and identification in higher layer will introduce a significant computational overhead [20].

There are a number of anti-eavesdropping counter-measures in WSNs. We roughly categorize them into three types: (i) lightweight encryption schemes [21,22,23,24]; (ii) generating artificial noise to limit the amount of information that can be extracted by eavesdroppers [25,26,27]; and (iii) mitigating the eavesdropping risk by controlling the transmitting power [28]. Table 1 summarizes these schemes. In particular, a number of lightweight encryption schemes based on physical layer features of wireless networks have been proposed [21,22,23,24]. The main idea of physical-layer encryption schemes is to exploit the inherent randomness of communication channels so that the amount of information that can be extracted by an eavesdropper is mitigated. However, the encryption schemes are still computational intensive and power-consuming.

Some recent studies [25,27] exploit the artificial noise generated by RFID readers to alleviate the eavesdropping capability of malicious nodes. However, these schemes can only be applied to the scenarios of Internet-of-Things (IoT) based on RFID. Besides, a transmitting power control method is proposed in [28] to mitigate the eavesdropping risk while to adjust the transmitting power may deteriorate the legitimate communications [29].

Although [15,16,17] also proposed an approach similar to our Fri-Jam schemes, their methods are mainly designed for the IoT scenario, in which jammers are placed outside the fence surrounding the boundary of the network. These schemes are not feasible to WSNs since eavesdroppers can appear at any location in WSNs. Besides, most of the studies on protective jamming schemes [15,16,17] are mainly focused on a specific scenario.

## 3. System Models

This section first presents three kinds of Fri-Jam schemes in Section 3.1. Then, Section 3.2 gives the channel model used in this paper. Section 3.3 presents the definitions on eavesdropping probability and transmission probability.

### 3.1. Fri-Jam Schemes

In this paper, we assume that the network is placed in a torus [30]. In this manner, the border effect can be ignored. We consider three types of users in our network: legitimate users, eavesdroppers and friendly jammers. The legitimate users are distributed according to homogeneous Poisson point process (PPP). Legitimate users transmit data packets, which might be passively snooped by eavesdroppers while legitimate users are unaware of the reconnaissance. Similar to [28], we assume that the eavesdropper is located at the center of the network without loss of generality since the network is placed in a symmetric torus.

**Figure 1:**
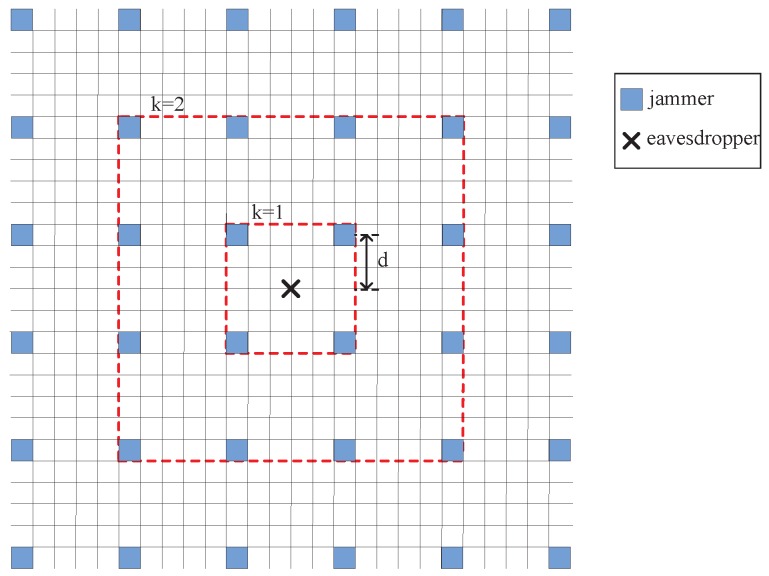
FJ-Reg Scheme: every jammer is placed at a gray square. Note that we only show a part of the whole network.

**Figure 2:**
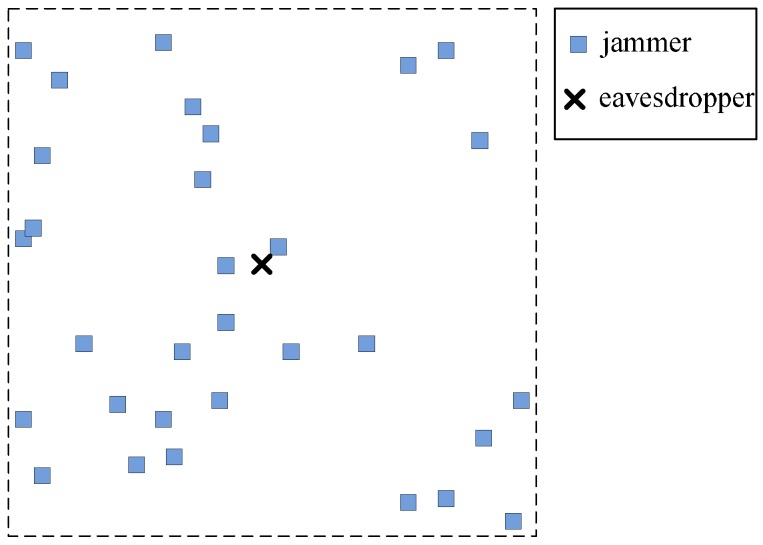
FJ-Ran Scheme: every jammer is randomly placed according to homogeneous Poisson Point Process (PPP). Note that we only show a part of the whole network.

The interference caused by friendly jammers heavily depends on the location of jammers and the emitting power of each jammer. In this paper, we consider two placement strategies of friendly jammers in WSNs: (i) FJ-Reg scheme, in which jammers are regularly placed at deterministic locations and (ii) FJ-Ran scheme, in which jammers are regularly placed at random locations. Specifically, in the FJ-Reg scheme, friendly jammers are regularly placed in a grid manner, as shown in Figure 1. In the FJ-Ran scheme, friendly jammers are randomly distributed according to according to PPP, as shown in Figure 2. In addition to FJ-Reg and FJ-Ran schemes, we also consider adjusting the emitting power of jammers. In particular, we consider a modified FJ-Reg scheme with power control (named FJ-PC scheme) in this paper.

### 3.2. Channel Model

We assume that the radio channel experiences Rayleigh fading and path loss. The received power of a receiver (i.e., a legitimate user or an eavesdropper) at a distance *r* from its nearest transmitter (legitimate user or friendly jammer) is $hr^{-\alpha }$, where *h* is a random variable following an exponential distribution with mean $\frac{1}{\mu }$ and *α* is the path loss factor. More specifically, we denote h∼exp(μ).

We then consider the *Signal to Interference plus Noise Ratio* (SINR) model. The SINR of the receiver at a random distance *r* from its transmitter is expressed as

(1) $$\mathrm{SINR}=\frac{P_{t}hr^{-\alpha }}{\sigma^{2}+I_{t}+I_{j}},$$

where $\sigma^{2}$ is the noise power, $I_{t}=\sum_{i\in \Phi /t_{0}}P_{t}h_{i}R_{i}^{-\alpha }$ denotes the cumulative interference from all the legitimate users except for the transmitter denoted by $t_{0}$, Φ denotes the set of legitimate users, $P_{t}$ denotes the transmitting power of the legitimate transmitter and $I_{j}$ denotes the cumulative interference generated by friendly jammers. The value of $I_{j}$ heavily depends on the placements of friendly jammers, which will be analyzed in Section 4.

We then define the *eavesdropping condition* to determine whether the transmission from a certain legitimate user can be wiretapped by an eavesdropper.

**Definition** **1.** *Eavesdropping Condition. A confidential transmission can be snooped by an eavesdropper if and only if SINR>T, where T is the received power threshold that an eavesdropper can successfully decode the transmission*.

### 3.3. Problem Definition

Based on the eavesdropping condition, we then define the *eavesdropping probability* denoted by P(E) as follows.

**Definition** **2.** *Eavesdropping Probability is the probability that at least one transmission has been wiretapped by an eavesdropper*.

From the definition we know P(E) is the probability to show how likely is any transmission eavesdropped. In order to derive P(E), we need to calculate the probability that one transmission has been eavesdropped, which is denoted by $P_{e}$. Considering the situation that no transmission being eavesdropped will be easier than considering all the situations that a certain number of transmissions being eavesdropped. Then, we find that P(E) can be expressed by $P_{e}$ as follows,

(2) $$P(E)=1-{\left(1-P_{e}\right)}^{N},$$

where *N* is the expected number of legitimate users in the network.

Another concern of this paper is to investigate the impacts of our Fri-Jam schemes on the legitimate communications. Thus, we define the transmission probability denoted by P(C) as follows.

**Definition** **3.** *Transmission Probability is the probability that a legitimate user (transmitter) can successfully transmit with another legitimate user (receiver)*.

To ensure the legitimate transmission, we require SINR>β at the legitimate receiver, where *β* is the threshold value of the receiving power for a successful reception. Thus, we have $P(C)\overset{\Delta }{\;=}P(\mathrm{SINR}>\beta )$. Following a similar approach to [31], we can obtain P(C).

## 4. Analysis on Eavesdropping Probability

We first present the analytical results on the eavesdropping probability of Non-Jam scheme in Section 4.1 and then present the results on the eavesdropping probability of Fri-Jam schemes in Section 4.2.

### 4.1. Analysis of Non-Jam Scheme

According to the definition of the eavesdropping probability P(E), we need to derive the probability $P_{e}$ that one transmission has been eavesdropped first. In particular, we have $P_{e}$ of Non-Jam scheme as follows.

**Theorem** **1.** *In Non-Jam scheme, the eavesdropping probability $P_{e}$ that one transmission has been eavesdropped is*

(3) $$P_{e}=\int_{r>0}e^{-\mu T_{p}r^{\alpha }\sigma^{2}-\pi r^{2}\lambda \left(\rho \left(T,\alpha \right)+1\right)}2\pi \lambda rdr,$$

*where $\rho \left(T_{p},\alpha \right)=T_{p}^{-2/\alpha }\int_{T_{p}^{-2/\alpha }}^{\infty }\frac{1}{1+\mu^{\alpha /2}}d\mu$ and $T_{p}=\frac{T}{Pt}$ for simplicity.*

**Proof.**  We denote the distance between the eavesdropper and its nearest transmitter by *r*. Since the transmitters are distributed according to PPP, the probability density function (PDF) of *r* can be derived as the following steps.

Firstly, we have the probability that no transmitter is closer than *R* given by

$$P\left[r>R\right]=P\left[\mathrm{No}\;\;\;\mathrm{transmitter}\;\;\mathrm{closer}\;\;\mathrm{than}\;\;R\right]=e^{-\lambda \pi R^{2}}.$$

Then, the cumulative distribution function (CDF) of *r* is $P\left[r\leq R\right]=F_{R}\left(R\right)=1-e^{-\lambda \pi R^{2}}$. We next have the PDF of *r* as follows,

$$f_{r}\left(r\right)=\frac{dF_{r}\left(r\right)}{dr}=e^{-\lambda \pi r^{2}}2\pi \lambda r.$$

Since the channel gain is *h*, the SINR at eavesdropper is

(4) $$\mathrm{SINR}=\frac{P_{t}hr^{-\alpha }}{\sigma^{2}+I_{t}},$$

where $I_{t}=\sum_{i\in \Phi /t_{0}}P_{t}h_{i}R_{i}^{-\alpha }$.

Then, the eavesdropping probability $P_{e}$ that one transmission has been eavesdropped is

(5) $$\begin{array}{cc} P_{e}= & E_{r}\left[P\left(\mathrm{SINR}>T|r\right)\right]\\ = & \int_{r>0}P\left[\frac{P_{t}hr^{-\alpha }}{\sigma^{2}+I_{t}}>T|r\right]e^{-\lambda \pi r^{2}}2\pi \lambda rdr\\ = & \int_{r>0}P\left[h>T_{p}r^{\alpha }\left(\sigma^{2}+I_{t}\right)|r\right]e^{-\lambda \pi r^{2}}2\pi \lambda rdr. \end{array}$$

Since *h* is a random variable following an exponential distribution with mean $\frac{1}{\mu }$, the probability becomes

(6) $$\begin{array}{cc} P[h>T_{p}r^{\alpha }\left(\sigma^{2}+I_{t}\right)|r]= & E_{I_{t}}\left[P\left[h>T_{p}r^{\alpha }\left(\sigma^{2}+I_{t}\right)|r\right]\right]\\ = & E_{I_{t}}\left[\exp\left[-\mu T_{p}r^{\alpha }\left(\sigma^{2}+I_{t}\right)\right]|r\right]\\ = & e^{-\mu T_{p}r^{\alpha }\sigma^{2}}\cdot E_{I_{t}}\left[\exp\left(-\mu T_{p}r^{\alpha }I_{t}\right)\right]\\ = & e^{-\mu T_{p}r^{\alpha }\sigma^{2}}\cdot L\left(\mu T_{p}r^{\alpha }\right), \end{array}$$

where L(·) denotes the Laplace transform.

More specifically, we have

$$\begin{array}{cc} L_{I_{t}}\left(s\right)= & E_{I_{t}}\left[e^{-sI_{t}}\right]\\ = & E_{\Phi ,\{h_{i}\}}\left[\exp(-s\sum_{i\in \Phi /b_{0}}h_{i}R_{i}^{-\alpha })\right]\\ = & E_{\Phi }\left[\prod_{i\in \Phi /b_{0}}\frac{\mu }{\mu +sR_{i}^{-\alpha }}\right]\\ = & \exp\left(-2\pi \lambda \int_{r}^{\infty }(1-\frac{\mu }{\mu +sv^{-\alpha }})vdv\right). \end{array}$$

Replacing variable *μ* with ${\left(\frac{v}{r{T_{p}}^{1/\alpha }}\right)}^{2}$, we then have

(7) $$L\left[\mu T_{p}r^{\alpha }\right]=\exp\left(-\pi r^{2}\lambda \rho \left(T_{p},\alpha \right)\right),$$

where $\rho \left(T_{p},\alpha \right)=T_{p}^{-2/\alpha }\int_{T_{p}^{-2/\alpha }}^{\infty }\frac{1}{1+\mu^{\alpha /2}}d\mu$. ☐

It is shown in Theorem 1 that the eavesdropping probability $P_{e}$ heavily depends on the channel conditions (such as the path loss and Rayleigh fading).

### 4.2. Analysis of Fri-Jam Schemes

Recall that we consider three Fri-Jam schemes: FJ-Reg, FJ-Ran and FJ-PC schemes. Thus, we categorize our analysis into the following cases.

#### 4.2.1. Case I: FJ-Reg Scheme

We first analyze the case of FJ-Reg, in which all the jammers are regularly placed in grid manner as shown in Figure 1. We denote the expectation of the cumulative interference generated by jammers by $E[I_{j}]$, which is given by Lemma 1.

**Lemma** **1.** *The expectation of the cumulative interference of regular placed jammers is*

(8) $$E\left[I_{j}\right]=\frac{1}{\mu }\sum_{m=1}^{n}E[I_{j}\left(m\right)].$$

We present the proof in Appendix A.

We then derive the probability $P_{e}$ that one transmission has been eavesdropped, which is given by Theorem 2.

**Theorem** **2.** *In FJ-Reg scheme, the probability $P_{e}$ that one transmission has been eavesdropped is*

(9) $$P_{e}=\int_{r>0}e^{-\mu T_{p}r^{\alpha }\left(\sigma^{2}+E[I_{j}\right)-\pi r^{2}\lambda \left(\rho \left(T_{p},\alpha \right)+1\right)}2\pi \lambda rdr,$$

*where $\rho \left(T_{p},\alpha \right)=T_{p}^{-2/\alpha }\int_{T_{p}^{-2/\alpha }}^{\infty }\frac{1}{1+\mu^{\alpha /2}}d\mu$ and $E[I_{j}]$ is given by Equation (8).*

**Proof.**  First, the SINR at a random distance *r* from its nearest transmitter can be expressed as $\mathrm{SINR}\;=\;\frac{hr^{-\alpha }}{\sigma^{2}+I_{t}+I_{j}}$. Then, from the definition of $P_{e}$, we have

$$\begin{array}{cc} P_{e}= & \int_{r>0}P\left[\frac{hr^{-\alpha }}{\sigma^{2}+I_{t}+I_{j}}>T_{p}|r\right]e^{-\lambda \pi r^{2}}2\pi \lambda rdr\\ = & \int_{r>0}P\left[h>T_{p}r^{\alpha }\left(\sigma^{2}+I_{t}+I_{j}\right)|r\right]e^{-\lambda \pi R^{2}}2\pi \lambda rdr. \end{array}$$

According to the channel model (given in Section 3.2), we have

$$\begin{array}{cc} P[h> & T_{p}r^{\alpha }\left(\sigma^{2}+I_{t}+I_{j}\right)\left|r\right]\\ = & E_{I_{t}}\left[\exp\left(-\mu T_{p}r^{\alpha }\right)\left(\sigma^{2}+I_{t}+I_{j}\right)|r\right]\\ = & e^{-\mu T_{p}r^{\alpha }\left(\sigma^{2}+E\left[I_{j}\right]\right)}\cdot E_{I_{t}}\left[\exp\left(-\mu T_{p}r^{\alpha }I_{t}\right)\right]\\ = & e^{-\mu T_{p}r^{\alpha }\left(\sigma^{2}+E\left[I_{j}\right]\right)}\cdot L\left(\mu T_{p}r^{\alpha }\right), \end{array}$$

where $L\left(\mu T_{p}r^{\alpha }\right)=\exp\left(-\pi r^{2}\lambda \rho \left(T_{p},\alpha \right)\right)$, $\rho \left(T_{p},\alpha \right)=T_{p}^{-2/\alpha }\int_{T_{p}}^{\infty }\frac{1}{1+{\left(\mu \right)}^{\alpha /2}}d\mu$ and $E[I_{j}]$ is given by Equation (8). ☐

It is shown in Theorem 2 that the probability $P_{e}$ heavily depends on the path loss factor *α*, the Rayleigh fading factor *μ*, the noise *σ* and the placement parameter *d*. Section 5 will give the numerical results that will further confirm this observation.

#### 4.2.2. Case II: FJ-Ran Scheme

We then analyze the case of FJ-Ran, in which all the jammers are randomly distributed in the network. Recall that both jammers and legitimate users are distributed according to PPP while they have the different distribution parameters. In particular, we denote the density of legitimate users by $\lambda_{1}$ and the density of friendly jammers by $\lambda_{2}$. Based on the well-known stochastic geometric results [31], we can obtain Theorem 3 on the probability $P_{e}$ that one transmission has been eavesdropped as follows.

**Theorem** **3.** *In FJ-Ran scheme, the probability $P_{e}$ that one transmission has been eavesdropped is*

$$\begin{array}{c} P_{e}=\int_{r>0}e^{-\mu T_{p}r^{\alpha }\sigma^{2}}\cdot L_{I_{t}}\left(\mu T_{p}r^{\alpha }\right)\cdot L_{I_{j}}\left(\mu T_{p}r^{\alpha }\right)e^{-\lambda_{1}\pi R^{2}}2\pi \lambda_{1}rdr, \end{array}$$

*where $L_{I_{t}}\left[\mu T_{p}r^{\alpha }\right]=\exp\left(-\pi r^{2}\lambda_{1}\rho \left(T_{p},\alpha \right)\right)$, $L_{I_{j}}\left[\mu T_{p}r^{\alpha }\right]=\exp\left(-\pi r^{2}\lambda_{2}\rho \left(T_{p},\alpha \right)\right)$ and $\rho \left(T_{p},\alpha \right)=T_{p}^{-2/\alpha }\int_{T_{p}^{-2/\alpha }}^{\infty }\frac{1}{1+\mu^{\alpha /2}}d\mu$.*

**Proof.**  According to the channel model defined in Section 3.2, we have the

(10) $$\begin{array}{cc} P_{e}= & \int_{r>0}P\left[\frac{P_{t}hr^{-\alpha }}{\sigma^{2}+I_{t}+I_{j}}>T|r\right]e^{-\lambda \pi r^{2}}2\pi \lambda rdr\\ = & \int_{r>0}P\left[h>T_{p}r^{\alpha }\left(\sigma^{2}+I_{t}+I_{j}\right)|r\right]e^{-\lambda_{1}\pi R^{2}}2\pi \lambda_{1}rdr. \end{array}$$

Following the similar analysis procedure to [31], we then have

(11) $$P\left[h>T_{p}r^{\alpha }\left(\sigma^{2}+I_{t}+I_{j}\right)|r\right]=e^{-\mu T_{p}r^{\alpha }\sigma^{2}}\cdot L_{I_{t}}\left(\mu T_{p}r^{\alpha }\right)\cdot L_{I_{j}}\left(\mu T_{p}r^{\alpha }\right).$$

Substituting $P[h>T_{p}r^{\alpha }\left(\sigma^{2}+I_{t}+I_{j}\right)|r]$ in Equation (10) by RHS of Equation (11), we finally prove the above result. ☐

It is shown in Theorem 3 that the probability $P_{e}$ heavily depends on both the node density $\lambda_{1}$ of legitimate users and the node density $\lambda_{2}$ of jammers, and the channel conditions.

#### 4.2.3. Case III: FJ-PC Scheme

We next analyze the case of FJ-PC scheme, in which jammers are placed in grid as the same as FJ-Reg scheme. We then assign the emitting power of jammers according to the different layers (as shown in Figure 1). We denote the layer number by *k*, which is ranging from 1 to *n*. The emitting power of jammers at the same layer is assigned with the same value. Specifically, we assign the emitting power at jammers in FJ-Reg scheme according to the following rule.

**Property** **1.** *We assign the emitting power of jammers at the kth layer according to the following equation:*

(12) $$P_{j}\left(k\right)=P_{J}\cdot \zeta^{1-k},$$

*where $P_{J}$ is the transmitting power of the jammers at the first layer and ζ is the power control factor.*

In FJ-PC scheme, the transmission probability of a legitimate user cannot be derived directly by using the existing approaches in [31,32,33,34] since the cumulative interference from jammers in FJ-PC scheme is quite different from that in FJ-Reg scheme. In particular, we have the following lemma to calculate the average cumulative interference.

**Lemma** **2.** *In FJ-PC scheme, the average cumulative interference from power controlled jammers to a legitimate transmitter is*

(13) $$E\left[I_{c}\right]=\frac{\sum_{k=1}^{n}\left(I_{k,t_{0}}+I_{k,t_{k}}+2\sum_{t=t_{1}}^{t_{k-1}}I_{k,t_{x}}\right)}{\sum_{k=1}^{n}2k},$$

*where $I_{k,t_{x}}$ is the interference at $t_{x}$, which can be calculated by*

(14) $$\begin{array}{cc} I_{k,t_{x}}= & \sum_{m=1}^{k}\sum_{v=1}^{m}\left(2P_{j}\left(m\right)\left({\sqrt{{\left[\left(v-\frac{1}{2}+t_{x}\right)\cdot 2d\right]}^{2}+{\left[\left(k-m+\frac{1}{2}\right)\cdot 2d\right]}^{2}}}^{-\alpha }\right.\right.\\ & +\left.\left.{\sqrt{{\left[\left(v-\frac{1}{2}+t_{x}\right)\cdot 2d\right]}^{2}+{\left[\left(k+m-\frac{1}{2}\right)\cdot 2d\right]}^{2}}}^{-\alpha }\right)\right)\\ & +\sum_{m=1}^{k}\sum_{w=k-m+1}^{k+m}2P_{j}\left(m\right){\sqrt{{\left[\left(m-\frac{1}{2}+t_{x}\right)\cdot 2d\right]}^{2}+{\left[\left(w-\frac{1}{2}\right)\cdot 2d\right]}^{2}}}^{-\alpha }\\ & +\sum_{q=k}^{n}\sum_{z=q-k-1}^{q+k}2P_{j}\left(q\right){\sqrt{{\left[\left(q-\frac{1}{2}+t_{x}\right)\cdot 2d\right]}^{2}+{\left[\left(z+\frac{1}{2}\right)\cdot 2d\right]}^{2}}}^{-\alpha }\\ & +\sum_{q=k}^{n}\sum_{s=1}^{q}\left(2P_{j}\left(q\right)\left({\sqrt{{\left[\left(s-\frac{1}{2}+t_{x}\right)\cdot 2d\right]}^{2}+{\left[\left(q-k-\frac{1}{2}\right)\cdot 2d\right]}^{2}}}^{-\alpha }\right.\right.\\ & +\left.\left.{\sqrt{{\left(\left(s-\frac{1}{2}+t_{x}\right)\cdot 2d\right)}^{2}+{\left[\left(q+k+\frac{1}{2}\right)\cdot 2d\right]}^{2}}}^{-\alpha }\right)\right) \end{array}$$

We present the proof in Appendix B.

We then derive the transmission probability P(C) of a legitimate user, which is given by Theorem 4.

**Theorem** **4.** *In FJ-PC scheme, the transmission probability P(C) is*

(15) $$P(C)=\int_{r>0}e^{-\mu \beta_{p}r^{\alpha }\left(\sigma^{2}+E\left[I_{c}\right]\right)-\pi r^{2}\lambda \left(\rho \left(\beta_{p},\alpha \right)+1\right)}2\pi \lambda rdr,$$

*where $\rho \left(\beta_{p},\alpha \right)={\beta_{p}}^{-2/\alpha }\int_{{\beta_{p}}^{-2/\alpha }}^{\infty }\frac{1}{1+\mu^{\alpha /2}}d\mu$, $\beta_{p}=\frac{\beta }{Pt}$ and $E[I_{c}]$ is given by Equation (13).*

**Proof.**  The SINR of the receiver at a random distance *r* from its nearest transmitter can be expressed as $\mathrm{SINR}=\frac{P_{t}hr^{-\alpha }}{\sigma^{2}+I_{t}+I_{c}}$, where $I_{c}$ is the cumulative interference caused by power controlled jammers on the recevier. Then, from the definition of P(C), we have

$$\begin{array}{cc} P(C)= & \int_{r>0}P\left[\frac{P_{t}hr^{-\alpha }}{\sigma^{2}+I_{t}+I_{c}}>\beta |r\right]e^{-\lambda \pi r^{2}}2\pi \lambda rdr\\ = & \int_{r>0}P\left[h>\beta_{p}r^{\alpha }\left(\sigma^{2}+I_{t}+I_{c}\right)|r\right]e^{-\lambda \pi R^{2}}2\pi \lambda rdr. \end{array}$$

According to the channel model (given in Section 3.2), we have

$$\begin{array}{cc} P[h> & \beta_{p}r^{\alpha }\left(\sigma^{2}+I_{t}+I_{c}\right)\left|r\right]\\ = & E_{I_{t}}\left[\exp\left(-\mu \beta_{p}r^{\alpha }\right)\left(\sigma^{2}+I_{t}+I_{c}\right)|r\right]\\ = & e^{-\mu \beta_{p}r^{\alpha }\left(\sigma^{2}+E\left[I_{c}\right]\right)}\cdot E_{I_{t}}\left[\exp\left(-\mu \beta_{p}r^{\alpha }I_{t}\right)\right]\\ = & e^{-\mu \beta_{p}r^{\alpha }\left(\sigma^{2}+E\left[I_{c}\right]\right)}\cdot L\left(\mu \beta_{p}r^{\alpha }\right), \end{array}$$

where $L\left(\mu \beta_{p}r^{\alpha }\right)=\exp\left(-\pi r^{2}\lambda \rho \left(\beta_{p},\alpha \right)\right)$, $\rho \left(\beta_{p},\alpha \right)={\beta_{p}}^{-2/\alpha }\int_{\beta_{p}}^{\infty }\frac{1}{1+{\left(\mu \right)}^{\alpha /2}}d\mu$ and $E[I_{j}]$ is given by Equation (8). ☐

We then have the eavesdropping probability $P_{e}$ in FJ-PC scheme as the following theorem.

**Theorem** **5.** *In FJ-PC scheme, the eavesdropping probability $P_{e}$ that one transmission has been eavesdropped is*

(16) $$P_{e}=\int_{r>0}e^{-\mu T_{p}r^{\alpha }\left(\sigma^{2}+E[{I_{j}}^{\prime }]\right)-\pi r^{2}\lambda \left(\rho \left(T_{p},\alpha \right)+1\right)}2\pi \lambda rdr,$$

*where $E\left[{I_{j}}^{\prime }\right]=E\left[4\sum_{k=1}^{n}P_{j}\left(k\right)\left(2{\left(d\cdot \sqrt{{\left(2k-3\right)}^{2}+{\left(2k-1\right)}^{2}}\right)}^{-\alpha }+{\left(d\cdot \sqrt{2{\left(2k-1\right)}^{2}}\right)}^{-\alpha }\right)\right]$.*

**Proof.**  The derivation of eavesdropping probability $P_{e}$ in FJ-PC scheme is similar to the derivation of Equation (15) in Theorem 4 and the main difference is the cumulative interference from jammers. In particular, the calculation of interference from *n*th layer jammers in FJ-PC scheme $I_{j}^{\prime }$ is similar to Equation (A3) in Appendix A, which is shown in the following equation:

(17) $$\begin{array}{ccc} I_{j}{\left(n\right)}^{\prime } & = & 4\sum_{k=1}^{n}P_{j}\left(k\right)\{2{\left(d\cdot \sqrt{{\left(2k-3\right)}^{2}+{\left(2k-1\right)}^{2}}\right)}^{-\alpha }\\ & & +{\left(d\cdot \sqrt{2{\left(2k-1\right)}^{2}}\right)}^{-\alpha }\}. \end{array}$$

Then we have the averaged cumulative interference from all the jammers as follows,

$$E\left[{I_{j}}^{\prime }\right]=E\left[\sum_{m=1}^{n}I_{j}{\left(m\right)}^{\prime }\right].$$

☐

According to the definition of the probability of eavesdropping attack P(E), we have

$$P\left(E\right)=1-{\left(1-P_{e}\right)}^{N},$$

where $P_{e}$ can be replaced by the different values as specified in Non-Jam scheme, FJ-Reg scheme, FJ-Ran scheme and FJ-PC Scheme, which can be obtained by Theorem 1, Theorem 2, Theorem 3 and Theorem 5, respectively. In the next section, we will present numerical results of P(E) based on the above schemes.

## 5. Numerical Results

In this section, we first present the numerical results of the probability of eavesdropping attacks P(E) with comparisons among different schemes in Section 5.1. Then we will show the impacts of friendly jammers on the legitimate communications in Section 5.2.

### 5.1. Comparisons of Different Schemes

**Figure 3:**
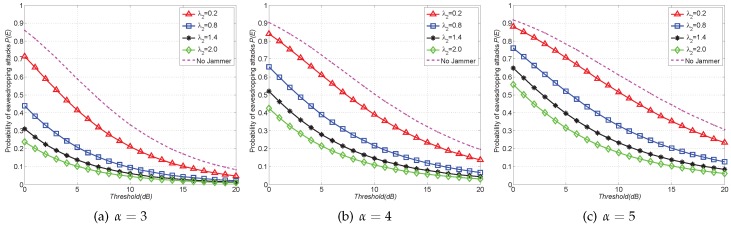
Probability of eavesdropping attacks P(E) with FJ-Ran scheme (PPP) versus Non-Jam scheme when α=3,4,5 with SINR threshold *T* ranging from 0dB to 20dB.

In the first set of results, we compare the probability of eavesdropping attacks P(E) of FJ-Ran scheme with that of Non-Jam scheme. Note that the larger node density $\lambda_{2}$ in FJ-Ran scheme and the smaller *d* in FJ-Reg scheme imply the higher cost (i.e., more jammers are deployed in the network). As shown in Figure 3, the results of Non-Jam scheme are shown in a dash curve and the results of FJ-Ran scheme are shown in solid curves with markers, where we choose the different values of node density $\lambda_{2}$ of friendly jammers (ranging from 0.2 to 2.0) and the value of node density $\lambda_{1}$ of legitimate user is 0.5. It is shown in Figure 3 that the Non-Jam scheme always has higher values of P(E) than the FJ-Ran scheme, implying that *using friendly jammers in WSN can effectively reduce the probability of eavesdropping attacks*.

It is also shown in Figure 3 that the probability of eavesdropping attacks P(E) decreases with the increment of jammers density $\lambda_{2}$, implying that *adding more jammers can further improve the effect of mitigating eavesdropping attacks*. For example, when α=4 and the threshold T=5dB (as shown in Figure 3b), P(E) of the Non-Jam scheme is 0.719 while P(E) of FJ-Ran scheme is reduced to 0.393 with jammers density $\lambda_{2}=0.8$ and 0.211 with jammers density $\lambda_{2}=2.0$.

**Figure 4:**
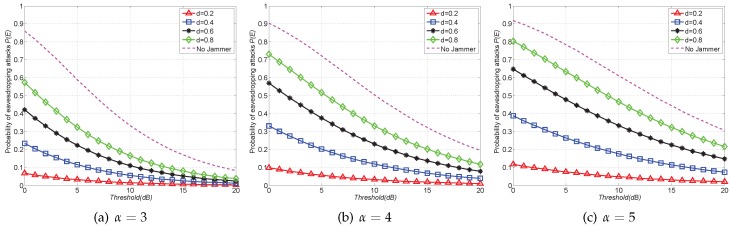
Probability of eavesdropping attacks P(E) with FJ-Reg scheme (Grid) versus Non-Jam scheme when α=3,4,5 with SINR threshold *T* ranging from 0dB to 20dB.

In the second set of results, we compare the probability of eavesdropping attacks P(E) of the FJ-Reg scheme with that of the Non-Jam scheme. Figure 4 shows the results, where a dash curve represents P(E) of the Non-Jam scheme and solid curves with markers depict the results of FJ-Reg scheme. Similar to Figure 3, we find that using friendly jammers can always reduce the eavesdropping probability compared with the Non-Jam scheme. Moreover, it is shown in Figure 4 that the probability of eavesdropping attacks P(E) heavily depends on both the channel conditions and system parameter *d*. Specifically, it is shown in Figure 4b that the probability of eavesdropping attack P(E) decreases with the decreased values of *d*. In fact, the *d* in FJ-Reg scheme plays a similar role to jammer density $\lambda_{2}$ in FJ-Ran scheme. In other words, decreasing *d* is equivalent to the effect of increasing jammer density $\lambda_{2}$. Take Figure 4b as an example again. When the threshold is T=5dB and α=4, P(E) of Non-Jam scheme is 0.7176 while P(E) becomes 0.072 with d=0.2, implying that using more friendly jammers can further reduce the eavesdropping probability.

**Figure 5:**
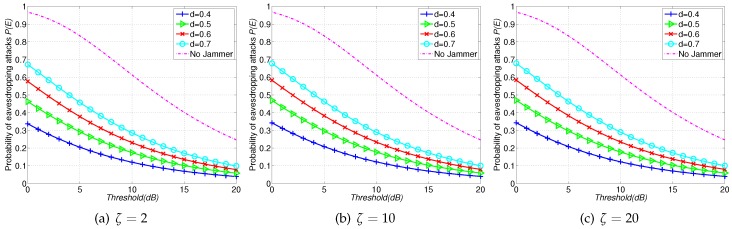
Probability of eavesdropping attacks P(E) with FJ-PC scheme versus Non-Jam scheme when ζ=2,10,20 with SINR threshold *T* ranging from 0dB to 20dB.

In the third set of results, we compare the probability of eavesdropping attacks P(E) of FJ-PC scheme with that of Non-Jam scheme. Figure 5 shows the results, where a dash curve represents P(E) of Non-Jam scheme and solid curves with markers depict the results of FJ-PC scheme. Similar to Figure 3 and Figure 4, we find that using friendly jammers can always reduce the eavesdropping probability compared with the Non-Jam scheme. Furthermore, we also find that the FJ-PC scheme can further reduce the eavesdropping probability compared with FJ-Reg scheme. This is due to the power assigning strategy in our FJ-PC scheme. In particular, the eavesdropping capability of the eavesdropper is significantly weakened by the jammers in the first layer, which have been assigned with higher power as they are much closer to the eavesdropper than other jammers in other layers. Another benefit of the FJ-PC scheme is that it has less impairment to legitimate communications compared with FJ-Reg and FJ-Ran schemes. The following results will further confirm this observation.

### 5.2. Impacts of Friendly Jammers on Legitimate Transmissions

Another concern is to investigate whether friendly jammers will significantly affect the legitimate transmissions. In order to differentiate the effect with jammers and the effect without jammers in terms of the eavesdropping probability and the transmission probability, we define the *eavesdropping deviation* $D_{E}$ and the *transmission deviation* $D_{C}$ as follows.

**Definition** **4.** Eavesdropping deviation $D_{E}$ is equal to the difference between the eavesdropping probability P(E) without jammers and the eavesdropping probability P(E) with jammers.

**Definition** **5.** Transmission deviation $D_{C}$ is equal to the difference between the transmission probability P(C) without jammers and the transmission probability P(C) with jammers.

We then derive the eavesdropping deviation $D_{E}$ and the transmission deviation $D_{C}$ in the first case of comparing FJ-Ran scheme with Non-Jam scheme. In particular, we have $D_{E}\left(\mathrm{Ran}\right)=P_{\mathrm{Non}-\mathrm{Jam}}\left(E\right)-P_{\mathrm{FJ}-\mathrm{Ran}}\left(E\right)$, where $P_{\mathrm{Non}-\mathrm{Jam}}\left(E\right)$ denotes the eavesdropping probability of Non-Jam scheme and $P_{\mathrm{FJ}-\mathrm{Ran}}\left(E\right)$ denotes the eavesdropping probability of FJ-Ran scheme. Besides, we have $D_{C}\left(\mathrm{Ran}\right)=P_{\mathrm{Non}-\mathrm{Jam}}\left(C\right)-P_{\mathrm{FJ}-\mathrm{Ran}}\left(C\right)$, where $P_{\mathrm{Non}-\mathrm{Jam}}\left(C\right)$ denotes the transmission probability of Non-Jam scheme and $P_{\mathrm{FJ}-\mathrm{Ran}}\left(C\right)$ denotes the transmission probability of FJ-Ran scheme. Note that P(C) can be calculated by [31] and we omit the detailed derivations in this paper.

Table 2 shows the comparison results. As shown in Table 2, the eavesdropping deviation is much larger than the transmission deviation at the same network settings, implying that *using jammers in WSNs will not significantly affect the legitimate communications* compared with the reductions on the eavesdropping probability. For example, when $\lambda_{2}=2.0$, $D_{E}=0.5178$ while there is less than 0.1 reduction on the transmission probability (i.e., $D_{C}=0.0963$).

Similarly, we derive the eavesdropping deviation $D_{E}$ and the transmission deviation $D_{C}$ in the second case of comparing FJ-Reg scheme with Non-Jam scheme. Table 3 shows the comparison results. It is shown in Table 3 that FJ-Reg scheme can also significantly reduce the eavesdropping probability with only minor influence on the legitimate transmissions (e.g., the reduction of P(E) is 0.6650 while the reduction of P(C) is only 0.1143 when d=0.2).

We next derive the eavesdropping deviation $D_{E}$ and the transmission deviation $D_{C}$ in the third case of comparing the FJ-PC scheme with the Non-Jam scheme. Table 4 shows the comparison results. It is shown in Table 4 that the FJ-PC scheme can significantly reduce the eavesdropping probability with only minor influence on the legitimate transmissions. For example, the P(E) is 0.6128 and P(c) is 0.1729 in the Non-Jam scheme and they become 0.1770 and 0.1367, respectively when FJ-PC scheme with d=0.6 is applied. At this time, the reduction of P(E) is 61.8% while the reduction of P(C) is only 12.5% when d=0.6 implying that the FJ-PC scheme can significantly reduce the eavesdropping probability while maintaining the minor impairment to the legitimate communications.

## 6. Conclusions

Wireless sensor networks (WSNs) are serving as a crucial component in cyber-physical social sensing systems. The security of WSNs has received extensive attention recently. One of the serious security threats in WSNs is eavesdropping attacks. In this paper, a novel anti-eavesdropping scheme has been proposed to alleviate eavesdropping attacks in WSNs. In particular, we deploy a number of friendly jammers that emit artificial noise to mitigate the eavesdropping capability of adversaries. More specifically, we consider three types of jamming schemes, such as regular placement of jammers (FJ-Reg), random placement of jammers (FJ-Ran) and regular placement of jammers with power control (FJ-PC). We establish a theoretical model to evaluate the performance of these jamming schemes. Our results show that to introduce friendly jammers in WSNs can significantly reduce the eavesdropping probability without the significant influence on the legitimate communications with the appropriate placement of jammers and the proper assignment of emitting power of jammers.

## Figures and Tables

**Table 1:** Comparison of related anti-eavesdropping schemes in WSNs.

	Encryption	Artificial Noise	Power Control
References	[8,9,10,11,18,21,22,23,24]	[25,26,27]	[28]
Limitations	computational intensive and power consuming	too specific (only apply for some specific scenarios)	deteriorate legitimate communications

**Table 2:** Eavesdropping deviation and transmission deviation of comparing FJ-Ran scheme with Non-Jam scheme when T=10dB and α=4.

Density $\lambda_{2}$	Eavesdropping deviation $D_{E}\left(\mathrm{Ran}\right)$	Transmission deviation $D_{C}\left(\mathrm{Ran}\right)$
0.2	0.1120	0.0303
0.8	0.3316	0.0718
1.4	0.4470	0.0880
2.0	0.5178	0.0963

**Table 3:** Eavesdropping deviation and transmission deviation of comparing FJ-Reg scheme with Non-Jam scheme when T=10dB and α=4.

Distance d	Eavesdropping deviation $D_{E}\left(\mathrm{Reg}\right)$	Transmission deviation $D_{C}\left(\mathrm{Reg}\right)$
0.2	0.6650	0.1143
0.4	0.5195	0.0977
0.6	0.3467	0.0742
0.8	0.2054	0.0500

**Table 4:** Eavesdropping deviation and transmission deviation of comparing FJ-PC scheme with Non-Jam scheme when T=10dB, ζ=10 and α=4.

Distance d	Eavesdropping deviation $D_{E}\left(\mathrm{PC}\right)$	Transmission deviation $D_{C}\left(\mathrm{PC}\right)$
0.4	0.4909	0.0594
0.5	0.4358	0.0362
0.6	0.3788	0.0217
0.7	0.3234	0.0132

## Appendix A

**Proof of** **Lemma 1.** We consider a coordinate system that is centered at the eavesdropper as shown in Figure 1. Since jammers are placed in a grid, each friendly jammer is 2d away from its nearest neighbor in the same axis. The transmitting power of each jammer is assumed to be $P_{J}$, which is same as the transmitting power of legitimate transmitters. From the channel model defined in Section 3.2, the radio signal received at an eavesdropper experiences both Rayleigh fading and the path loss. We consider the path loss effect first and then extend our analysis with consideration of Rayleigh fading effect.

We first calculate the cumulative interference emitted from jammers at the 1st layer, which is shown as follows,

$$I_{j}\left(1\right)=4P_{J}{\left(\sqrt{2}d\right)}^{-\alpha }.$$

Similarly, we have the interference from jammers at the 2nd layer as follows,

$$I_{j}\left(2\right)=4P_{J}\left[2{\left(\sqrt{10}d\right)}^{-\alpha }+{\left(3\sqrt{2}d\right)}^{-\alpha }\right].$$

The interference from jammers at the 3rd layer is given by

$$I_{j}\left(2\right)=4P_{J}\left\{2\cdot \left[{\left(\sqrt{10}d\right)}^{-\alpha }+{\left(3\sqrt{2}d\right)}^{-\alpha }\right]+{\left(5\sqrt{2}d\right)}^{-\alpha }\right\}.$$

Following the similar analysis, we have the interference from jammers at the (n−1)-th layer as follows,

(A1) $$\begin{array}{c} \begin{array}{cc} I_{j}\left(n-1\right) & =4P_{J}\{2\cdot [{\left(d\cdot \sqrt{1+{\left(2n-3\right)}^{2}}\right)}^{-\alpha }\\ & +\;{\left(d\cdot \sqrt{9+{\left(2n-3\right)}^{2}}\right)}^{-\alpha }+\cdots \\ & +\;{\left(d\cdot \sqrt{{\left(2n-5\right)}^{2}+{\left(2n-3\right)}^{2}}\right)}^{-\alpha }]\\ & +\;{\left(d\cdot \sqrt{2{\left(2n-3\right)}^{2}}\right)}^{-\alpha }\}. \end{array} \end{array}$$

Then, the interference from the *n*-th layer is given by

(A2) $$\begin{array}{c} \begin{array}{cc} I_{j}\left(n\right) & =4P_{J}\{2\cdot [{\left(d\cdot \sqrt{1^{2}+{\left(2n-1\right)}^{2}}\right)}^{-\alpha }\\ & +\;{\left(d\cdot \sqrt{9+{\left(2n-1\right)}^{2}}\right)}^{-\alpha }+\cdots \\ & +\;{\left(d\cdot \sqrt{{\left(2n-3\right)}^{2}+{\left(2n-1\right)}^{2}}\right)}^{-\alpha }]\\ & +\;{\left(d\cdot \sqrt{2{\left(2n-1\right)}^{2}}\right)}^{-\alpha }\}. \end{array} \end{array}$$

Summarizing them all, we then have

(A3) $$\begin{array}{c} I_{j}\left(n\right)=4P_{J}\sum_{k=1}^{n}\left\{2{\left(d\cdot \sqrt{{\left(2k-3\right)}^{2}+{\left(2k-1\right)}^{2}}\right)}^{-\alpha }+{\left(d\cdot \sqrt{2{\left(2k-1\right)}^{2}}\right)}^{-\alpha }\right\}. \end{array}$$

We next have the cumulative interference from all the jammers as follows,

$$I_{j}=\sum_{m=1}^{n}I_{j}\left(m\right).$$

Considering the Rayleigh fading effect, we finally prove the expectation the cumulative interference from all the jammers as given in Lemma 1. ☐

## Appendix B

**Proof of** **Lemma 2.** We assume that the transmitter is located between *k*th layer and k+1th layer of the jammers. Since the jammers are distributed in a symmetric manner (around the eavesdropper), we only need to calculate the cumulative interference from one corner of the plane (e.g., the north-east corner). Summing the cumulative interference from all the four corners, we can obtain the cumulative inference from all the jammers.

Firstly, we consider the interference caused from friendly jammers at the center location between 1st layer and 2nd layer as shown in Figure 1 (see the red dashed line). Interference caused by jammers at the *m*th layer, which is surrounded by the jammers at the *k*th layer is calculated as the following equation,

(B1) $$\begin{array}{cc} I_{j}\left(m\right) & =P_{j}\left(m\right)\{2{\sqrt{{\left(\frac{1}{2}\cdot 2d\right)}^{2}+{\left[\left(k-m+\frac{1}{2}\right)\cdot 2d\right]}^{2}}}^{-\alpha }\\ & +2{\sqrt{{\left(\frac{3}{2}\cdot 2d\right)}^{2}+{\left[\left(k-m+\frac{1}{2}\right)\cdot 2d\right]}^{2}}}^{-\alpha }+\cdots \\ & +2{\sqrt{{\left[\left(m-\frac{1}{2}\right)\cdot 2d\right]}^{2}+{\left[\left(k-m+\frac{1}{2}\right)\cdot 2d\right]}^{2}}}^{-\alpha }\\ & +2{\sqrt{{\left[\left(m-\frac{1}{2}\right)\cdot 2d\right]}^{2}+{\left[\left(k-m+\frac{3}{2}\right)\cdot 2d\right]}^{2}}}^{-\alpha }+\cdots \\ & +2{\sqrt{{\left[\left(m-\frac{1}{2}\right)\cdot 2d\right]}^{2}+{\left[\left(k+m-\frac{1}{2}\right)\cdot 2d\right]}^{2}}}^{-\alpha }\\ & +2{\sqrt{{\left[\left(m-\frac{3}{2}\right)\cdot 2d\right]}^{2}+{\left[\left(k+m-\frac{1}{2}\right)\cdot 2d\right]}^{2}}}^{-\alpha }+\cdots \\ & +2{\sqrt{{\left(\frac{1}{2}\cdot 2d\right)}^{2}+{\left[\left(k+m-\frac{1}{2}\right)\cdot 2d\right]}^{2}}}^{-\alpha }\}. \end{array}$$

Summing all the terms in Equation (B1), we then obtain the simplified expression of $I_{j}\left(m\right)$ as follows,

(B2) $$\begin{array}{cc} I_{j}\left(m\right) & =2P_{j}\left(m\right)\{\sum_{v=1}^{m}\left({\sqrt{{\left[\left(v-\frac{1}{2}\right)\cdot 2d\right]}^{2}+{\left[\left(k-m+\frac{1}{2}\right)\cdot 2d\right]}^{2}}}^{-\alpha }\right.\\ & +\left.{\sqrt{{\left[\left(v-\frac{1}{2}\right)\cdot 2d\right]}^{2}+{\left[\left(k+m-\frac{1}{2}\right)\cdot 2d\right]}^{2}}}^{-\alpha }\right)\\ & +\sum_{w=k-m+1}^{k+m}{\sqrt{{\left[\left(m-\frac{1}{2}\right)\cdot 2d\right]}^{2}+{\left[\left(w-\frac{1}{2}\right)\cdot 2d\right]}^{2}}}^{-\alpha }\}. \end{array}$$

Interference caused by jammers at the *q*th layer, which is placed outside of *k*th layer is calculated by this equation,

(B3) $$\begin{array}{cc} I_{j}\left(q\right) & =P_{j}\left(q\right)\{2{\sqrt{{\left(\frac{1}{2}\cdot 2d\right)}^{2}+{\left[\left(q-k-\frac{1}{2}\right)\cdot 2d\right]}^{2}}}^{-\alpha }\\ & +2{\sqrt{{\left(\frac{3}{2}\cdot 2d\right)}^{2}+{\left[\left(q-k-\frac{1}{2}\right)\cdot 2d\right]}^{2}}}^{-\alpha }+\cdots \\ & +2{\sqrt{{\left(\left(q-\frac{1}{2}\right)\cdot 2d\right)}^{2}+{\left[\left(q-k-\frac{1}{2}\right)\cdot 2d\right]}^{2}}}^{-\alpha }\\ & +2{\sqrt{{\left(\left(q-\frac{1}{2}\right)\cdot 2d\right)}^{2}+{\left[\left(q-k+\frac{1}{2}\right)\cdot 2d\right]}^{2}}}^{-\alpha }+\cdots \\ & +2{\sqrt{{\left(\left(q-\frac{1}{2}\right)\cdot 2d\right)}^{2}+{\left[\left(q+k+\frac{1}{2}\right)\cdot 2d\right]}^{2}}}^{-\alpha }+\cdots \\ & +2{\sqrt{{\left(\frac{1}{2}\cdot 2d\right)}^{2}+{\left[\left(q+k+\frac{1}{2}\right)\cdot 2d\right]}^{2}}}^{-\alpha }\}. \end{array}$$

From Equation (B3) we can get the simplified expression of $I_{j}\left(q\right)$ as follows,

(B4) $$\begin{array}{cc} I_{j}\left(q\right) & =2P_{j}\left(q\right)\{\sum_{s=1}^{q}\left({\sqrt{{\left(\left(s-\frac{1}{2}\right)\cdot 2d\right)}^{2}+{\left[\left(q-k-\frac{1}{2}\right)\cdot 2d\right]}^{2}}}^{-\alpha }\right.\\ & +\left.{\sqrt{{\left(\left(s-\frac{1}{2}\right)\cdot 2d\right)}^{2}+{\left[\left(q+k+\frac{1}{2}\right)\cdot 2d\right]}^{2}}}^{-\alpha }\right)\\ & +\sum_{z=q-k-1}^{q+k}{\sqrt{{\left(\left(q-\frac{1}{2}\right)\cdot 2d\right)}^{2}+{\left[\left(z+\frac{1}{2}\right)\cdot 2d\right]}^{2}}}^{-\alpha }\}. \end{array}$$

Therefore, summing Equations (B2) and (B4), we obtain the cumulative interference of jammers at the location $t_{0}$ as the following equation,

(B5) $$\begin{array}{cc} I_{k,t_{0}} & =\sum_{m=1}^{k}\sum_{v=1}^{m}\left(2P_{j}\left(m\right)\left({\sqrt{{\left[\left(v-\frac{1}{2}\right)\cdot 2d\right]}^{2}+{\left[\left(k-m+\frac{1}{2}\right)\cdot 2d\right]}^{2}}}^{-\alpha }\right.\right.\\ & +\left.\left.{\sqrt{{\left[\left(v-\frac{1}{2}\right)\cdot 2d\right]}^{2}+{\left[\left(k+m-\frac{1}{2}\right)\cdot 2d\right]}^{2}}}^{-\alpha }\right)\right)\\ & +\sum_{m=1}^{k}\sum_{w=k-m+1}^{k+m}2P_{j}\left(m\right){\sqrt{{\left[\left(m-\frac{1}{2}\right)\cdot 2d\right]}^{2}+{\left[\left(w-\frac{1}{2}\right)\cdot 2d\right]}^{2}}}^{-\alpha }\\ & +\sum_{q=k}^{n}\sum_{z=q-k-1}^{q+k}2P_{j}\left(q\right){\sqrt{{\left[\left(q-\frac{1}{2}\right)\cdot 2d\right]}^{2}+{\left[\left(z+\frac{1}{2}\right)\cdot 2d\right]}^{2}}}^{-\alpha }\\ & +\sum_{q=k}^{n}\sum_{s=1}^{q}\left(2P_{j}\left(q\right)\left({\sqrt{{\left[\left(s-\frac{1}{2}\right)\cdot 2d\right]}^{2}+{\left[\left(q-k-\frac{1}{2}\right)\cdot 2d\right]}^{2}}}^{-\alpha }\right.\right.\\ & +\left.\left.{\sqrt{{\left[\left(s-\frac{1}{2}\right)\cdot 2d\right]}^{2}+{\left[\left(q+k+\frac{1}{2}\right)\cdot 2d\right]}^{2}}}^{-\alpha }\right)\right) \end{array}$$

The interference at $t_{x}$ can be calculated by the similar approach in Equation (14).

We denote the number of all the possible locations of jammers placed between *k*th layer and (k+1)-th layer as $N_{k}$, which is equal to ${\left(2k+1\right)}^{2}-{\left(2k-1\right)}^{2}=8k$. Finally, the averaged interference is calculated by taking an average over all the possible locations. The detailed calculation is shown as the following equation,

(B6) $$E\left[I_{c}\right]=\frac{\sum_{k=1}^{n}\left(4I_{k,t_{0}}+4I_{k,t_{k}}+8\sum_{t=t_{1}}^{t_{k-1}}I_{k,t}\right)}{\sum_{k=1}^{n}8k}=\frac{\sum_{k=1}^{n}\left(I_{k,t_{0}}+I_{k,t_{k}}+2\sum_{t=t_{1}}^{t_{k-1}}I_{k,t}\right)}{\sum_{k=1}^{n}2k}.$$

☐
